# Combined Interstitial and Intracavitary High-Dose Rate Brachytherapy of Cervical Cancer

**DOI:** 10.3389/fonc.2021.809825

**Published:** 2022-01-13

**Authors:** Jun Itami, Naoya Murakami, Miho Watanabe, Shuhei Sekii, Takahiro Kasamatsu, Shingo Kato, Hisako Hirowatari, Hitoshi Ikushima, Ken Ando, Tatsuya Ohno, Hiroyuki Okamoto, Kae Okuma, Hiroshi Igaki

**Affiliations:** ^1^ Shin-Matsudo Accuracy Radiation Therapy Center, Shin-Matsudo Central General Hospital, Chiba, Japan; ^2^ Department of Radiation Oncology, National Cancer Center Hospital, Tokyo, Japan; ^3^ Department of Radiology, Chiba University Graduate School of Medicine, Chiba, Japan; ^4^ Department of Radiation Oncology, Hyogo Prefectural Cancer Center, Hyogo, Japan; ^5^ Department of Gynecology, Tokyo Metropolitan Bokuto Hospital, Tokyo, Japan; ^6^ Department of Radiation Oncology, International Medical Center, Saitama Medical University, Saitama, Japan; ^7^ Department of Radiation Oncology, Tokyo Rinkai Hospital, Tokyo, Japan; ^8^ Department of Radiation Oncology, Tokushima University Faculty of Medicine, Tokushima, Japan; ^9^ Department of Radiation Oncology, Gunma Cancer Center, Gunma, Japan; ^10^ Department of Radiation Oncology, Gunma University Graduate School of Medicine, Gunma, Japan; ^11^ Department of Medical Physics, National Cancer Center Hospital, Tokyo, Japan

**Keywords:** cervical cancer, interstitial, intracavitary, brachytherapy, three-dimensional image-guided

## Abstract

High-dose-rate brachytherapy by remote afterloading is now performed under three-dimensional image guidance by CT or MRI. Three-dimensional image-guided brachytherapy in cervical cancer disclosed that the traditional intracavitary brachytherapy by Manchester method cannot deliver an adequate dose to the large tumor with resulting local recurrence. To improve the local control rate, combined interstitial and intracavitary (hybrid) brachytherapy can increase the dose to the large parametrial involvement without increasing the dose to the rectum and bladder. Whether hybrid brachytherapy can be performed safely on a multi-institutional basis remains to be studied. From 2015, phase I/II study of hybrid brachytherapy was launched in Japan, and it was revealed that hybrid brachytherapy can be performed safely and with a high quality of radiation dose distribution in a multi-institutional study. In Japan, the number of patients undergoing hybrid brachytherapy in cervical cancer is rapidly rising. Education and clinical trial are very important to establish hybrid brachytherapy in the management of cervical cancer.

## Image-Guided Brachytherapy of the Cervical Cancer

Brachytherapy has been applied mainly in head and neck cancers, cutaneous cancers, gynecological cancers, and prostate cancers. Currently, most brachytherapy is performed in a high-dose rate (HDR) because HDR brachytherapy does not require a radiation-shielded ward and enables image-guided optimization easily. In cervical cancer, intracavitary brachytherapy (ICBT) has been demonstrated to play a decisive role in curative radiation therapy (1). Even the most precise external beam radiation therapy (EBRT) like intensity-modulated radiation therapy (IMRT) and stereotactic radiation therapy cannot replace brachytherapy without lowering progression-free and overall survivals (2). ICBT of cervical cancer uses tandem and ovoid applicators. In ICBT, the dose has been prescribed traditionally to an imaginary point A in the paracervical triangle according to the Manchester method (3). Orthogonal x-rays can locate the point A that lies 2 cm lateral to the intrauterine tandem at the point 2 cm cranial from the external os. Point A is anatomically corresponding to the point where the uterine artery crosses the ipsilateral ureter. However, precise anatomical relationships between the tumor, the organs at risk (OARs), and point A is not obvious with orthogonal x-rays.

Three-dimensional image-guided brachytherapy (3D-IGBT) has been applied in clinical practice since around 2000, where CT or MRI of the treated regions is performed to clarify the 3D anatomy of the gross tumor volume (GTV) and the OARs using CT/MRI compatible applicators in the treatment position (4). With the introduction of 3D-IGBT, it has become clear that a large tumor often does overgrow point A, and brachytherapy dose prescribed to point A is not enough to encompass the large tumor (5, 6). Some tumors recur in the low-dose region lying out of point A. MRI has been shown to play a decisive role in 3D-IGBT of cervical cancer because of an excellent soft-tissue contrast for delineation of anatomical structures (1, 4, 7). According to the EMBRACE studies, high-risk clinical target volume (HR-CTV) of brachytherapy is defined as the residual tumor just before ICBT plus the cervix that are revealed by T2 weighted images (T2WI) of MRI, and ICBT should deliver boost dose to this HR-CTV up to more than 85 Gy in equivalent dose assumed to be delivered by 2 Gy fractions (EQD_2_) including the dose from EBRT (4, 5, 8). Therefore, MRI with the applicators *in situ* is used as a gold standard in the EMBRACE studies. In contrast, 3D-IGBT using CT has a problem of difficulty in differentiating soft tissue contrasts and HR-CTV extracted by CT with the applicators *in situ* is usually larger than the HR-CTV delineated by T2WI (9). However, many institutions have difficulties in using MRI for each brachytherapy session due to logistic issues and the situation is especially serious in Japan, where less than 3% of the institutions performing HDR-ICBT are routinely using MRI for each ICBT session (10). CT is a much-preferred modality to be used in radiation oncology in many countries other than in Europe. Studies have shown that with a help of diagnostic MRI without the applicators *in situ*, HR-CTV can be contoured consistently by CT, and consensus guidelines for CT delineation of HR-CTV at the time of brachytherapy have been published (9, 11). Additionally, meta-analyses have disclosed the superiority of 3D-IGBT compared with two-dimensional brachytherapy planning with point A in local control and morbidities (12, 13).

## Hybrid Brachytherapy of the Cervical Cancer

**Figure 1:**
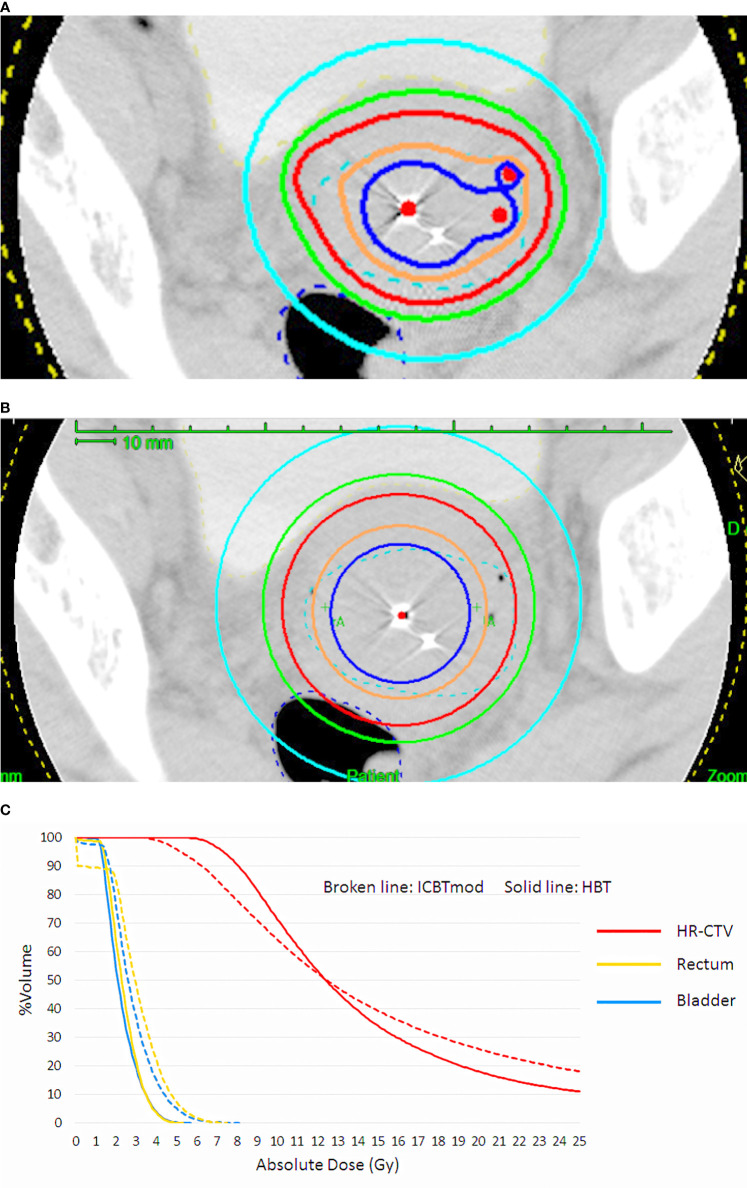
**(A)** Dose distribution of hybrid brachytherapy (HBT) and **(B)** dose distribution of intracavitary irradiation with increasing dwell times of ovoid applicator (ICBTmod) to cover high-risk CTV with D90 ≥ 6 Gy. **(C)** Dose-volume histogram (DVH) shows increased rectal and bladder doses in ICBTmod. HBT can deliver an adequate high-risk CTV dose without increment of rectal and bladder dose.

**Figure 2:**
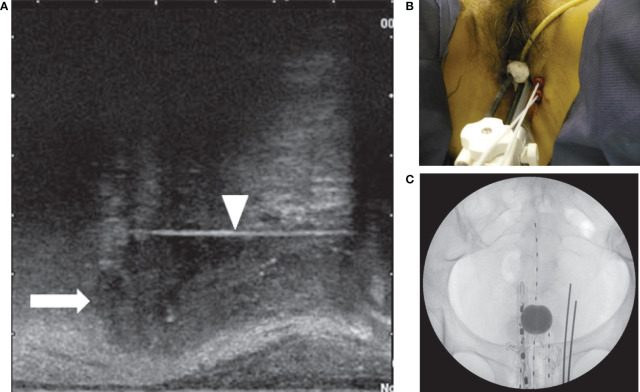
**(A)** Sagittal view of the transrectal ultrasound showing the parametrial invasion (arrow) and the interstitial perineal applicator (arrowhead). The applicator is inserted up to the cranial margin of the parametrial invasion. **(B)** Two perineal interstitial applicators were inserted to the left of intracavitary applicators. **(C)** Anterior x-ray of intracavitary and interstitial applicators. Also seen is a rectal dosimeter.

**Figure 3:**
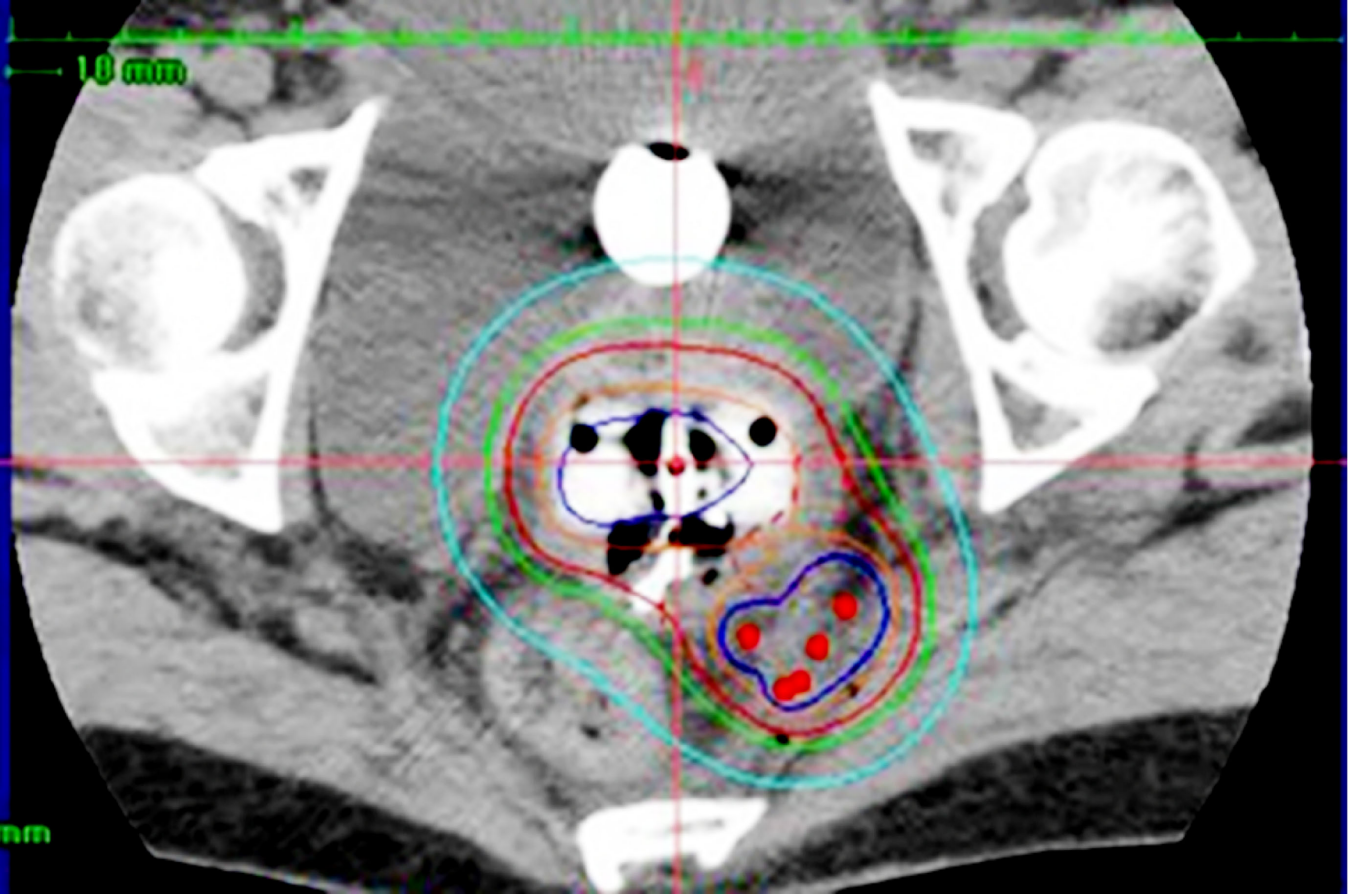
Hybrid brachytherapy of cervical cancer extending along the left uterosacral ligament. Five red dots posterolateral to the intracavitary applicators are perineal interstitial applicators, with which good coverage of the uterosacral invasion is attained.

With the EMBRACE studies, the long-standing controversy of radiation oncology was solved whether a boost radiation dose should be applied to the initially involved tumor volume or the shrunken residual tumor volume. Boost dose is now administered to the residual tumor volume, represented by HR-CTV. It is possible to raise the dose to the large residual cervical tumor by increasing dwell times of the ipsilateral ovoid applicator in ICBT, despite the inevitable increments of rectal and bladder doses. As only 3 applicators are used in ICBT, the possibility of dose distribution modification is very limited. If interstitial applicators are inserted into the parametrial region lateral to point A in addition to the intracavitary applicators, a rise of tumor dose is possible without increasing the rectal and bladder doses (**Figure 1**) (4, 5, 14). Yoshida et al. have demonstrated by a simulation study that the tumor larger than 4 cm × 3 cm × 3 cm had better be treated by a combination of ICBT and interstitial brachytherapy (ISBT) [hybrid brachytherapy (HBT)], not by ICBT alone (15). Both vaginal and perineal approaches are available for the insertion of interstitial applicators into the parametrium. Specialized vaginal applicators suitable for transvaginal application were developed (16, 17) and commercially available. However, in Japan, these specialized applicators are not available and many institutions are doing free-hand transvaginal and/or perineal interstitial applications. The interstitial applicators are implanted under transrectal ultrasound guidance (**Figure 2**), and they are inserted up to the cranial margin of the parametrial tumor. The transvaginal application causes post-removal bleeding, although infrequently, therefore gauze pressure packing of the vaginal cavity is mandatory after the transvaginal application. The transperineal application can cover even the posterolateral tumor extension along the uterosacral ligament easily, which is difficult by transvaginal application (**Figure 3**).

## Clinical Trial of Hybrid Brachytherapy in Japan

**Figure 4:**
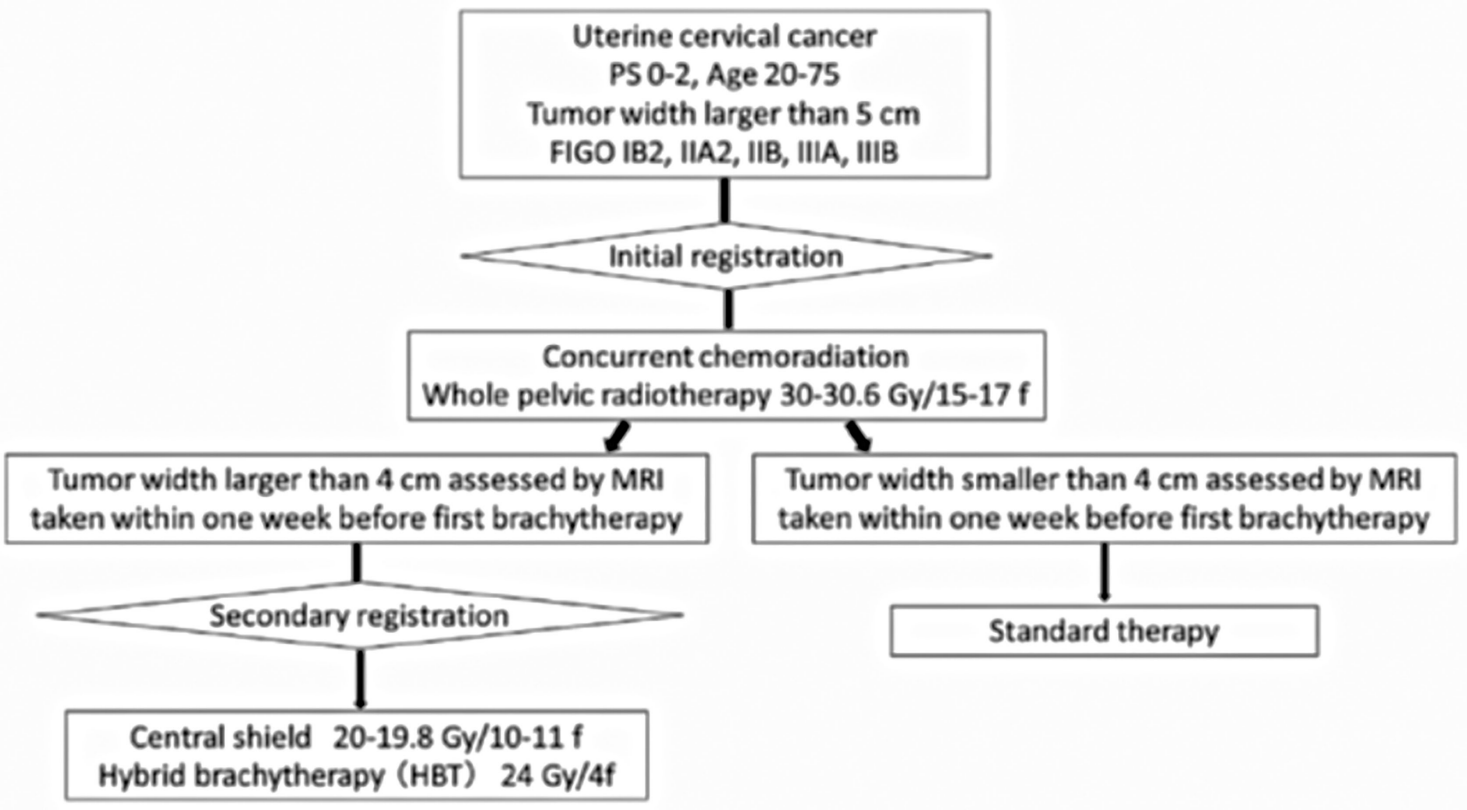
Shema of the phase I/II trial of hybrid brachytherapy.

While HBT appears to be superior to the ICBT alone in a large cervical cancer from the standpoint of dose distribution and local control (5), it remains to be studied whether HBT can be performed safely on a multi-institutional basis in Japan. In 2015, we launched the multi-institutional phase I/II clinical trial of HBT for locally advanced cervical cancer (18). Twenty leading institutions in Japan performing HDR brachytherapy of cervical cancer participated in the trial. The locally advanced cervical cancer with an initial diameter of more than 5 cm with T2WI was recruited as the first step (**Figure 4**). Patients with distant metastases were excluded. The registered patients underwent EBRT to the whole pelvis in a conventional fractionation with concurrent weekly cis-diaminedichloro-platinum (CDDP) of 40 mg/m^2^. If T2WI post-30 Gy EBRT disclosed a residual tumor larger than 4 cm, the patients were entered in the final registration. The finally registered patients were treated by EBRT to the bilateral pelvic sidewalls with a central pelvic shielding up to 50 Gy including the whole pelvis dose. In many Japanese institutions, central shielding is used routinely for IIIB patients after 30 Gy of the whole pelvis EBRT (19), therefore central shielding pelvic radiation therapy was employed as the EBRT in this study, and IMRT was not accepted. In Japan, currently, an increasing number of institutions are transitioning to IMRT from central shielding in order to increase the dose to the primary (20). HBT was administered with 6 Gy 4 times, twice weekly immediately after completion of the whole pelvic radiation therapy. The dose was prescribed to the HR-CTV with D90 ≥ 6 Gy/fraction. Therefore, if the tumor dose delivered by the centrally shielded pelvic radiation is ignored, the tumor EQD_2_ is only 62 Gy in contrast to the EMBRACE recommendations of more than 85 Gy in EQD_2_ (7). On the day of HBT, EBRT was not performed. A number of the interstitial applicators should be less than 7 with a hyperdose sleeve (21) around the interstitial applicator less than 1 cm in diameter. Other dose constraints of HBT + EBRT are shown in **Table 1** (7). Both MRI and CT are allowed to conduct 3D-IGBT, and the definition of contouring of HR-CTV was defined independently for this study (18). All 19 institutions but one used CT for each HBT session. If the parametrial tumor cannot be encompassed by 6 needle applicators with keeping hyperdose sleeves around the applicators of less than 1 cm, the patients were treated by extensive ISBT using more than 6 needles and excluded from the study. If the residual tumor was smaller than or equal to 4 cm by T2WI after 30 Gy of EBRT and CDDP, they are treated by usual ICBT out of protocol. In the phase I portion, 20 patients were planned to be entered in the final registration and it was analyzed whether the HBT can be performed safely on a multi-institutional basis with less than 10% incidence of ≥grade 3 acute nonhematological morbidities caused by HBT according to CTCAE ver. 4 (22). The detailed results will be published elsewhere, but till Feb. 2017, 20 patients were accrued to the phase I part of the study and only one patient had uterine bleeding from the transvaginal insertion site of grade 3 at the time of applicator removal. As the incidence of serious nonhematological morbidities was less than 10% (1/20, 5%), the study progressed to phase II part to see the efficacy of HBT. Till the study closure in Oct. 2019, 73 patients were recruited for the 1st registration and 52 patients (71% of the 1st registration) advanced to the final registration. Clinical characteristics of the patients are shown in **Table 2**. Although we had assumed that 50% of the 1st-step registrants progressed to the final step, tumor shrinkage after 30 Gy of EBRT and weekly CDDP was not so remarkable as assumed and even 71% of the patients went to the final step and underwent HBT. As for dose constraints of the rectum, bladder, and sigmoid, 98.1%, 100%, and 100% of the patients were irradiated per protocol, respectively. Quality of HBT was determined by HR-CTV D90 with 94.2% of the patient irradiated per protocol with a fractional HBT dose ≥6 Gy. From the phase I portion, we could conclude that HBT can be performed safely and with a satisfactory quality of radiation delivery on a multi-institutional basis in Japan. The primary endpoint of the study was 2-year intrapelvic control and the final results of this phase I/II trial will be published soon elsewhere.

**Table 1:** Dose constraints of HBT plus EBRT.

	Per protocol	Acceptable deviation
Rectum D_2 cc_ (Gy, EQD_2_, total dose)	≤75 Gy	Not applicable
Bladder D_2 cc_ (Gy, EQD_2_, total dose)	≤90 Gy	Not applicable
Sigmoid colon D_2 cc_ (Gy, EQD_2_, total dose)	≤75 Gy	Not applicable
HR-CTVD_90_ (Gy, physical dose, per each HBT)	≥6 Gy	≥5.4 Gy
Mean diameter of hyperdose sleeve around interstitial applicators (cm)	≤1 cm	≤1.5 cm

EBRT, external beam radiation therapy; EQD_2_, equivalent dose in 2 Gy fraction; HBT, hybrid brachytherapy; HR-CTV, high-risk clinical target volume.

**Table 2:** Characteristics of the patients recruited to the phase I/II trial.

	Primary registrants (*n* = 73)	Secondary registrants (*n* = 52)
PS		
0	52	37
1	21	15
2	0	0
Age		
Median (years, range)	48 (26–74)	48 (26–74)
FIGO Stage (2008)		
IB2	11	10
IIA2	4	2
IIB	29	20
IIIA	1	0
IIIB	28	20
	Primary registrants (*n* = 71^a^)	Secondary registrants (*n* = 52)
Tumor width before treatment		
Median (cm, range)	5.70 (4.30–9.20)	5.70 (4.30–9.20)
Pelvic lymph node metastasis		
No	36	29
Yes	35	23
Histopathology		
SCC	64	47
Adeno	6	4
Adenosquamous	1	1
Others	0	0

^a^Two patients retracted consent and their details are unknown.

Adeno, adenocarcinoma; Adenosquamous, adenosquamous carcinoma; FIGO, International Federation of Gynecology and Obstetrics; PS, performance status; SCC, squamous cell carcinoma.

## Increased Use of Hybrid Brachytherapy in the Cervical Cancer in Japan

**Figure 5:**
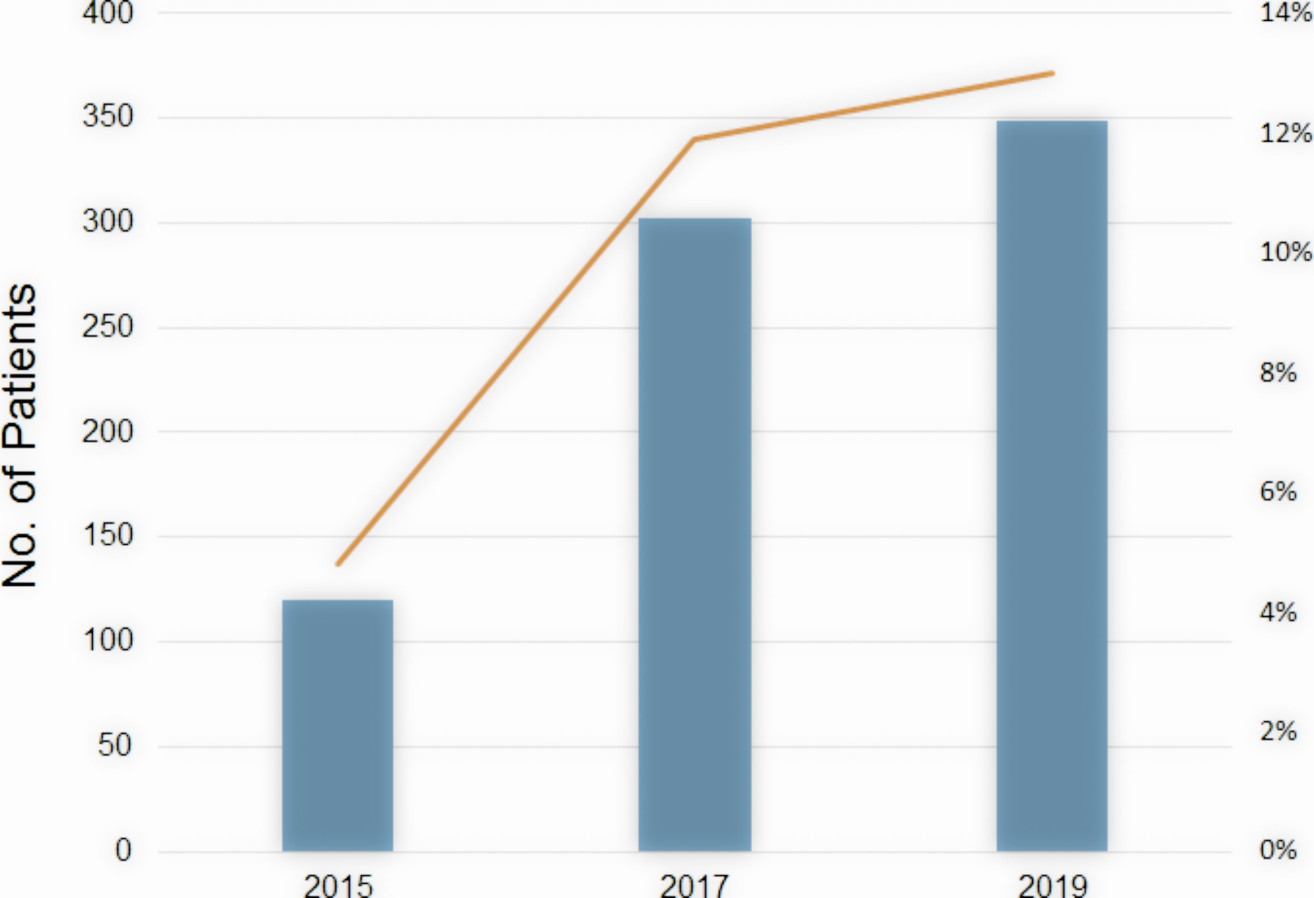
The number of patients undergoing HBT in Japan (bar) and the ratio of HBT in brachytherapy of cervical cancer (line) by year.

The number of patients undergoing HBT is rising rapidly in Japan. According to the MicroSelectron Research Group in Japan (personal communication) which covers more than 80% of radiation facilities using HDR remote afterloading devices in Japan, a number of patients with cervical cancer undergoing HBT were 120 patients in 2015, 302 in 2017, and 349 in 2019 (**Figure 5**). The ratio of HBT in brachytherapy of cervical cancer increases correspondingly from 4.8% in 2015 to 13% in 2019. Many radiation oncologists are now accustomed to HBT procedure which is more invasive than ICBT, and they are now implementing HBT to increase the dose to the involved parametria. Educational seminars held by the specialists in HBT and by the vendor as well as site-visiting training by coaching doctors of the HBT clinical trial group contributed to the spread of HBT in Japan as well. Many Japanese radiation oncologists now realize that HBT can be easily performed and can deliver an adequate dose to the large parametrial invasion without increasing the rectal and bladder doses.

## Conclusions

There is now abundant evidence that increased local control can be obtained with a larger dose in many cancer types (5, 14, 23, 24) and the HBT can deliver increased dose to cervical cancer without increasing doses to the OARs. An increasing number of institutions in Japan are now implementing HBT in the radiotherapy of locally advanced cervical cancer thanks to educational efforts. For the development of brachytherapy including HBT, hand-on education is mandatory. Furthermore, a multi-institutional randomized trial comparing ICBT vs. HBT is desired to establish the role of HBT in the radiotherapeutic management of cervical cancer.

## Author Contributions

Clinical trial planned by JI, NM, KO, and HO. MW, SS, TK, SK, HH, HIk, KA, TO and HIg were responsible investigators of the participating institutes.

## Funding

This work was supported by MHLW Research for Promotion of Cancer Control Programmes Grant Number 21EA1010.

## Conflict of Interest

The authors report research grant from Elekta and ITOCHU and consultation honoraria from HekaBio, Alpha-TAU, and Palette Science.

## Publisher’s Note

All claims expressed in this article are solely those of the authors and do not necessarily represent those of their affiliated organizations, or those of the publisher, the editors and the reviewers. Any product that may be evaluated in this article, or claim that may be made by its manufacturer, is not guaranteed or endorsed by the publisher.
